# A Comparative Review of Autologous Conditioned Serum and Autologous Protein Solution for Treatment of Osteoarthritis in Horses

**DOI:** 10.3389/fvets.2021.602978

**Published:** 2021-02-19

**Authors:** Livia Camargo Garbin, Michael J. Morris

**Affiliations:** Department of Veterinary Clinical Sciences, School of Veterinary Medicine, Faculty of Medical Sciences, The University of the West Indies at St. Augustine, St. Augustine, Trinidad and Tobago

**Keywords:** osteoarthritis, horse, autologous conditioned serum, IL-1Ra, autologous protein solution, growth factors, anti-inflammatory cytokines

## Abstract

Many alternative treatments aimed at modulating osteoarthritis (OA) progression have been developed in the past decades, including the use of cytokine inhibitors. IL-1β is considered one of the most impactful cytokines in OA disease and therefore, its blockage offers a promising approach for the modulation of OA. Interleukin-1 receptor antagonist (IL-1Ra) is a naturally occurring anti-inflammatory protein belonging to the IL-1 family that competes with IL-1β for occupancy of its receptors, without triggering the same downstream inflammatory response. Because of its natural anti-inflammatory properties, different methods have been proposed to use IL-1Ra therapeutically in OA. Autologous conditioned serum (ACS) and autologous protein solution (APS) are blood-derived products produced with the use of specialized commercial kits. These processes result in hemoderivatives with high concentrations of IL-1Ra and other cytokines and growth factors with potential modulatory effects on OA progression. Several studies have demonstrated potential anti-inflammatory effect of these therapies with promising clinical results. However, as with any hemoderivatives, clinical outcomes may vary. For optimal therapeutic use, further research is warranted for a more comprehensive understanding of the product's composition and interaction of its components in joint inflammation. Additionally, differences between ACS and APS treatments may not be clear for many clients and clinicians. Thus, the objective of this narrative review is to guide the reader in important aspects of ACS and APS therapies, *in vitro* and *in vivo* applications and to compare the use of both treatments in OA.

## Introduction

Osteoarthritis is a common cause of lameness observed in horses (1), and has been described as a disease with a common end stage of progressive degeneration of the articular cartilage (2). Subchondral bone and soft tissues are also affected (2). This disease may occur early in the career of equine athletes or later in older horses (3), being responsible for up to 60% of lameness cases (2, 4, 5).

Biological therapies such as platelet-rich plasma (PRP), autologous protein solution (APS), and autologous conditioned serum (ACS) provide a more physiological alternative to conventional treatments capable of modulating inflammation in OA (6–8). These products are derived from the patient's own blood and are rich in anti-inflammatory cytokines and growth factors that reduce inflammation and promote anabolism in tissues (7). These therapies have demonstrated significant clinical and histological improvement in horses with OA (8, 9). Although the proof-of-principle for hemoderivatives is widely known, there are still several limitations for its use. Patient's own biological variability, lack of standardization of the protocols for preparation and application are a few of these limitations. Those variables result in inconsistent concentration of bioactive factors leading to conflicting results (9–11). In addition, although APS and ACS are distinct hemoderivatives, their similarities in cytokine composition may be confusing. The objective of this review is to describe important aspects of ACS and APS therapies, to characterize these products and to compare their modulatory effect in osteoarthritis.

## Inflammation and Role of IL-1Ra in Osteoarthritis

The inflammatory process plays a paramount role in the pathogenesis of OA, involving the subchondral bone, cartilage and synovial tissues. These tissues release pro-inflammatory cytokines, as IL-1ß and TNF-α, which initiate and propagate inflammation (12). These cytokines promote synovial inflammation and lead chondrocytes to release metalloproteinases (MMP 1, 3, and 13), aggrecanases 4 and 5 (ADAMTS-4 and 5), reactive oxygen specie (ROS) and cytokines such as cyclooxygenase 2 (COX-2) (12). These, in addition to prostaglandin 2 (PGE_2_), IL-6 and IL-8 propagate OA (13–16), resulting in cartilage matrix degradation (17–19). Both IL-1β and TNF-α are increased in synovial fluid, synovial membrane, cartilage and subchondral bone during OA (12).

IL-1β binds to two different receptors: type I IL-1 receptor, which leads to downstream regular IL-1 activity; and the type II receptor which is a decoy receptor. Blockage of type I IL-1 receptor results in downregulation of MMPs, ADAMTS, and pro-inflammatory cytokines that propagate OA (20). IL-1Ra is a natural protein that antagonizes the effects of IL-1 by binding to both type I and II receptors without triggering the expected downstream effects of IL-1 (21, 22). IL-Ra is produced by many types of cells including chondrocytes, monocytes and synovial fibroblasts during inflammation (23, 24). In osteoarthritis, as well as other degenerative diseases, the concentration of IL-1Ra available in affected tissues is too low to inhibit the negative effects of IL-1. Different *in vivo* studies suggest that the concentration of IL-1Ra should be 10–1,000-fold higher than the concentration of IL-1 to effectively block IL-1 receptors with significant therapeutic effect (25). Since the discovery of IL-1Ra in 1986 (20), multiple methods of enhancing the concentration of this protein in hemoderivatives have been identified (26–28). The enhancement of endogenous IL-1Ra could be an efficient therapeutic approach in diseases where IL-1 plays an important role in the pathophysiology (22). Recent biochemical modulatory therapies aimed at restoring the anabolic-catabolic balance within the joint, (thereby modulating progression of OA), have been developed to increase IL-1Ra and other anti-inflammatory cytokines and improve clinical outcomes (21, 29).

## Autologous Conditioned Serum

The first autologous conditioned serum (ACS) preparation method (Orthokine®) (29), was developed and patented in 2003 by Peter Wehling and Julio Reinecke. This is the most used current method of up-regulating IL-1Ra from whole blood. Although this product has been slowly validated, it gained fast clinical acceptance primarily in human and veterinary sports medicine (20). Autologous conditioned serum is produced by incubating whole blood for 24 h at 37°C with medical grade glass beads coated with CrSO_4_, generating an enriched serum. After incubation, the blood clots and serum are centrifuged, collected and filtrated before administration (29).

During incubation, platelets degranulate and mononuclear cells are stimulated to produce various cytokines and growth factors such as; IL-1Ra, IL-4, TNF-α, IL-10, IL-6, and basic fibroblast growth factor (FGFb) among various other anabolic factors stated on Table 1 (27, 28, 35, 36). It was initially stated that this process would not result in the concomitant increase of pro-inflammatory cytokines as IL-1 ß and TNF-α (29). However, more recent studies showed that the concurrent increase in pro-inflammatory cytokines may happen in whole blood incubation (36).

**Table 1:** Comparison between autologous conditioned serum and autologous protein solution.

**Type of hemoderivative**	**Commercial kits**	**Amount of blood**	**Anticoagulant**	**Equipment necessary**	**Protocol**	**Platelet concentration**	**Leucocyte concentration**	**Growth-factors/** **anti-inflammatory** **cytokines**	**Pro-inflammatory cytokines**	**IL-Ra/IL-β**	**Number of treatments**	**References**
Autologous conditioned serum (ACS)	IRAP II® Ortokine®	60 mL	No anticoagulant used	Kit, incubator, centrifuge and freezer	Kit is incubated at 37°C for 24 h. Then, the kit is centrifuged for 10 min and serum is collected. Serum is stored at −20°C.	Not reported (30) or lower than whole blood (31)(not statistically significant)	Not reported (30) or lower than whole blood (31) (not statistically significant)	IGF-1, IL-10, sTNF-R1, TGF-β, IL-1Ra, IL-6, IL-4, FGFb,VEGF, HGF,PDGF-AB, osteoprotegerin, interferon-gamma, oncostatin M	TNF-α, IL-1 β, MMP-3	5 (30) to 113 (31)	4	(9, 30, 31)
Autologous protein solution (APS)	Pro-Stride® nSRIDE®	55 mL	ACD-A	Kit and centrifuge (separator and concentrator)	Blood is placed on the separator and centrifuged for 15 min and platelet-rich solution is collected. The product is transferred to the concentrator and centrifuged again for 2 min.	Higher than whole blood (31)	Higher than whole blood (31)	sTNF-R1, TGF-β, IL-1Ra, PDGF-AB/BB, IGF-1, EGF	TNF-α, IL-1β, MMP-3	48.22 (31)	1	(8, 31–34)

*sTNF-R1, soluble tumor necrosis factor receptor 1; TGF-β, transforming growth factor beta; FGFb, basic fibroblast growth factor; HGF, hepatocyte growth factor; IL-1Ra, interleukin 1 receptor antagonist protein; PDGF-AB/BB, platelet-derived growth factor AB/BB; IGF-1, insulin-like growth factor; EGF, epidermal growth factor; MMP-3, metalloproteinase 3; osteoprotegerin, osteoclastogenesis inhibitory factor; interferon, gamma, oncostantin M*.

Despite the aforementioned studies, the ratio between anti-inflammatory cytokines and pro-inflammatory cytokines (specifically IL-1Ra/IL-1β) should still be sufficient to justify therapeutic use (20). In fact, considering an IL-1Ra concentration of 3 ng/mL in an ACS kit (Orthokine®), 2 mL of Orthokine® can be injected into the patients' knee, resulting in a minimum ratio of IL-1Ra/IL-1β of 170:1. This is much higher than the minimum 10:1 ratio necessary to be clinically efficient (28).

Monocytes are believed to be responsible for the cytokine increase within the ACS, with both surface area and the chemical-cell interaction playing important role in the type of cytokine produced (20). With regard to surface area, Magalon et al. (37) demonstrated that bead diameter results in varied monocyte induction. For instance, beads of 3 mm and polished beads of 3.5 mm produced higher anti and pro-inflammatory cytokine concentrations compared with other diameters (4 and 2.5 mm coated with CrSO_4_) (37).

Different studies suggested that the chemical-cell interaction would be essential for cytokine expression in monocytes (28, 38). Initially, it was observed that cellulose acetate beads, used for adsorptive apheresis of granulocytes and monocytes, induced IL-1Ra release in peripheral blood with no concurrent release of TNF-α or IL1β (38).

Interestingly, anti-inflammatory cytokines and IL-1Ra/IL-1ß ratio increased in equine whole blood samples incubated in glass tubes without beads (30). This increase in cytokines was not statistically different compared with serum obtained from commercial kits (30). This indicates that CrSO_4_-treated glass beads may not be necessary to produce an enriched serum (37). On the other hand, in Meijer et al. (28), the incubation of whole blood with medical grade borosilicate beads did induce significant increase in IL-1Ra concentrations compared with standard consumer beads. Thus, as in any other biological therapies, the methods and kits used for ACS production, influence the end product cytokine profile, which could potentially impact its clinical properties. This also indicates that other cytokines present in ACS may play a role in the inflammatory modulation of this therapy (28).

The most common commercial kits require a period of 24 h for preparation of ACS. In those kits, the increased concentration of IL-1Ra is time-dependent, reaching a maximum concentration in 24 h (28). Prolonged periods of whole blood incubation does not increase concentrations of cytokines or growth factors in ACS (39). Notably, the use of certain medications e.g., reserpine and phenylbutazone have been demonstrated to interfere with platelet function and clotting, which may potentially interfere with ACS cytokine content (40, 41).

### Use of Autologous Conditioned Serum as a Therapy for Osteoarthritis

The therapeutic effects of IL-1Ra have been of interest to researchers as a potential modulatory treatment for OA. Numerous *in vitro* and *in vivo* studies involving different animal models have tested the efficacy of this therapy (9, 20, 30, 42, 43). Conditioned serum (CS) stimulated chondrogenic differentiation in adipose-derived stem cells and induced these cells to reduce lymphocyte proliferation and activation *in vitro* (44). It is possible that intra-articular CS administration influence resident stem cell behavior and immunomodulation (44). *In vivo*, IL-1Ra reduced osteophyte formation and cartilage fibrillation in rabbits (17).

In a randomized block study using an equine model, animals were injected at days 14, 21, 28, and 35 after OA induction and subsequently compared with placebo (9). A significant decrease of synovial proliferation and overall joint inflammation was observed in treated animals compared with placebo. Similar clinical improvement was observed in human patients with knee osteoarthritis (10). Still, ACS is yet to be proven to prevent cartilage degradation (9). The administration of ACS may stimulate the endogenous production of IL-1Ra, since this cytokine significantly increased over time after ACS injection (9). In another study, it was determined that natural inflammatory mediators such as IL-1β and IL-6 can potentially activate IL-1Ra transcription in chondrocytes and macrophages *in vitro* (23). The positive results obtained experimentally supported the clinical use of ACS in equine sports medicine (45).

## Autologous Protein Solution

Over a decade after the initial investigation of ACS (32), a newer hemoderivative called autologous protein solution (APS) was developed. This method proposes to increase the anti-inflammatory and anabolic concentrations of hemoderivatives of clinical use. Autologous protein solution consists of a platelet-rich plasma (PRP) separated from the blood. The PRP is then processed through a special kit intended to stimulate white blood cells (WBC) to produce anti-inflammatory cytokines concentrating its content in a smaller volume of plasma.

For the preparation of this product, blood is collected and mixed with anticoagulant citrate dextrose solution (ACD-A), transferred to a separation device and centrifuged for 15 min (8, 46). Then, the intermediate cell solution (containing platelets and white blood cells) is transferred to an APS concentrator, where the solution is mixed with polyacrylamide beads and centrifuged again for 2 min. The autologous protein solution is then collected and ready for use (32, 46).

In horses, APS presented significantly more white blood cells, platelets and significantly less erythrocytes compared with the whole blood (31). Different cytokines were detected in APS such as TNF-α, TGF-β, IL-1β, IL-6, IL-10, MMP-3, and IL-1Ra among others (Table 1) (8, 31, 33, 46). In fact, a positive ratio of IL1Ra:IL-1β was observed in APS for horses (31). Increased values of interleukin 1 receptor I and tumor necrosis factor receptor II (sIL-1RI and sTNF-RII; Table 1) have been observed in APS in humans (32).

Cytokines in APS seem to correlate to the cellular content within the product. For instance, a negative correlation has already been determined between WBC and TGF-β concentrations (31). Platelets may also negatively correlate to TGF- β and sTNF-1R (31). Interestingly though, a positive correlation between leukocytes and IL-1Ra concentrations, but not IL-1ß has also been observed (47).

The general condition of the patients did not seem to interfere significantly with the cytokine composition of APS. Similar concentrations of anti-inflammatory cytokines and anabolic growth factors were observed in APS from patients with OA and controls (32).

### Use of Autologous Protein Solution as a Therapy for Osteoarthritis

Although not extensively studied as other hemoderivatives, APS has been successfully used as a symptom modifying option for the treatment of OA. This treatment seems to be safe and its side-effects (i.e., discomfort post-injection) are commonly associated with other intraarticular injections (34).

The use of APS has provided optimistic results for OA treatment in different species (8, 48, 49). In a prospective, blinded, placebo-controlled clinical trial performed in dogs, animals presented improvement in pain, lameness assessment and weight bearing in the affected limb 12 weeks after APS treatment, compared with placebo treated joints (49).

In fact, one single injection of APS demonstrated significant improvement in Western Ontario and McMaster Universities osteoarthritis index (WOMAC) scores in OA patients after 2 weeks post-injection compared with baseline (34). Pain scores improved up to 90% in 6 months post-treatment (34). In addition to the clinical improvement, APS treatment reduced bone-marrow lesions 12 months post-treatment (50). However, results should be interpreted with caution since OA varied among the patients and some were actually medicated with non-steroidal anti-inflammatory drugs (NSAIDs), which may have influenced the results (50).

In horses with naturally occurring OA, APS significantly improved lameness, pain-in-flexion, gait analysis and range of motion up to 14 days after treatment compared with baseline and controls (8). In equine joint fluid, there was a significant decrease in protein concentration in treated horses compared to untreated controls (8).

Promising results with APS use were justified due to its favorable cytokine content. A IL-1Ra:IL-1 ratio >1,000 in APS seemed to predict the inhibition of inflammation in human OA patients (47). Additionally, IL-1Ra and sTNF-RII content in APS was correlated with improvement in pain scores after treatment (47). Whilst such correlations are still in their infancy, further studies, focusing on the integration of APS cytokines on the multiple inflammatory pathways in the different stages of OA, are warranted.

## Discussion

Although positive results were observed with clinical use of ACS and APS, others demonstrated that ACS did not have a direct effect on cartilage metabolism compared with unstimulated serum. This may justify the lack of disease modifying effects observed clinically with the use of these products (36, 42).

### Concentration of Cytokines in Joints After Treatment

The conflicting results observed *in vivo* may be attributed to the reduction of therapeutic cytokine concentrations in the synovial fluid after injection, suggesting that new intra-articular therapies should focus on the prolonged presence of the cytokines in the joint space. For this reason, some authors opted for a protocol of multiple serial ACS injections separated by short periods of time (42).

It is still unknown how long the cytokines remain present in the joint and to what extent they exercise an effect (20). In fact, most of the cytokines in ACS are released within 6 h and increased hours of incubation at body temperature are known to reduce bioactivity of these cytokines (36). For instance, IL-1Ra has a short half-life of only 4–6 h. Thus, it is uncertain if the IL-1Ra concentrations within the OA joints actually correlate with anti-inflammatory effects of ACS as the ratio of IL-1Ra/IL-1β in synovial fluid of patients has not been correlated with disability and pain (51). Similarly in horses, in joints treated with a single injection of APS, synovial concentrations of IL-1 β, TNF-α, and IL-1Ra were not significantly different compared with control groups (8). A standard protocol was suggested by Lasarzik et al. (52), which described better results with 2-day treatment interval for ACS injections. This was based on the evidence that in horses with OA, ACS induced significant decrease of IL-1β and increase of IL-1Ra in synovial fluid, despite these effects lasting no more than 48 h (52). With regards to APS use, this issue has not been raised and the protocols described, currently involved the use of only one intra-articular treatment (8, 53). Nevertheless, it might be interesting to study the effects of a single vs. multiple APS injections in the modulation of OA.

### Therapeutic Effect of Autologous Conditioned Serum and Autologous Protein Solution in Chronic Osteoarthritis

It is possible that the blockage of IL-1 receptors is insufficient to abolish cartilage degradation in more advanced cases of OA (11, 51, 54). Indeed, the use of ACS did not significantly delay or prevent surgical intervention in end-stage osteoarthritis in humans compared with placebo after 10 years of treatment (11). For APS, treatment did demonstrate significant clinical improvement up to 3 years after injection mainly in patients with mild to moderate knee OA (48). In horses, the use of APS was clinically more efficient when no severe lameness, asymmetry in gait or subchondral bone sclerosis was observed in arthritic joints (8). Animals were up to 30-times more likely to present therapeutic improvement with APS treatment when affected with mild OA (8). Similarly, in humans, patients with better cartilage conditions responded better compared with patients with more significant cartilage loss (11).

Such findings suggest that the inhibition of IL-1Ra in chronic cases of OA may not be sufficient to improve clinical outcome. In cases in which disease was identified early though, clinical improvement was reported (9, 42). Osteoarthritis and rheumatoid arthritis are complex inflammatory processes that involve multiple pathways and cytokines that change dynamically during the course of the condition (55, 56). Thus, the impact of hemoderivatives in these multiple pathways may need to be further explored (57).

### Modulatory Effects of Autologous Conditioned Serum and Autologous Protein Solution in Joint Tissues

The modulatory effects of ACS and APS therapies have been validated experimentally despite the lack of a comprehensive understanding of their effects on multiple pathways in OA (8, 9, 31, 48). A recent study suggested that both ACS and APS seems to have more efficient anti-inflammatory and chondroprotective effects compared with triamcinolone in an *in vitro* OA model. These hemoderivatives significantly reduced expression of IL-1ß and TNF-α and showed a trend in upregulation of aggrecan (ACAN) and collagen type II (COL2-A1) expression in cartilage (31). In the synovium, both treatments upregulated IL-10 gene expression and significantly decreased the release of PGE_2_ in synovium compared with triamcinolone (31). The effects of ACS and APS seemed to be dose dependent (31).

Studies such as Velloso Alvarez et al. (31), revealed overall similar effects of ACS and APS in a co-culture model, although the cytokine profile of these hemoderivatives is essentially different. The effects of the different cytokine profiles may be more easily observed in simpler models as monocultures (46). In chondrocytes, the concentrations of IL-1Ra and IL-10 released by cells treated with APS were actually higher than ACS treated cells, although no difference was observed in gene expression. The significant difference in cytokine release obtained in monocultures vs. co-culture models can indicate that the pool of cytokines in both hemoderivative products may influence tissues differently, but that the overall effect in joints may be similar.

Additionally, detailed investigation of the ACS cytokines other than IL-1Ra and their individual (or synergic) effect in different tissues within the joint, may reveal other potential treatment routes equally or more important than IL-1 blockage. For instance, in horses with mild osteoarthritis, clinical improvement was statistically associated with content of not only IL-1Ra but also IGF-1 in ACS (57). *In vivo* studies will be paramount to better establish the importance of such cytokine interactions and their relevance in a clinical scenario.

### Limitations and Requirements for Treatment Use

As with other biological therapies, the optimization of protocols for preparation and application of ACS and APS in different OA scenarios is warranted. Considering the autologous nature of the product, cytokine variability is an inevitable issue with this treatment, which can lead to inconsistent modulatory and clinical effects (57). The development of an allogeneic preparation of these products, as suggested for other hemoderivatives, could provide a more consistent cytokine profile (58).

Autologous protein solution currently offers a more user-friendly approach since it requires shorter preparation and no incubation times, unlike ACS which requires a minimum laboratory setting (i.e., centrifuge, incubator, and freezer). Autologous protein solution can be prepared using a portable centrifugation equipment and is a very basic and undemanding technique (8). The intra-articular injections can be applied as a quick, point-of -care single treatment in an ambulatory-based practice (8). Conversely, in case the practitioner is already familiar with the ACS technique and equipment, the continuation of its use would be pertinent. There is insufficient evidence-based research to support superiority of APS compared with ACS at this point.

## Final Considerations

Despite the limited number of clinical studies using ACS and APS and the lack of knowledge in relation to its composition, these therapies provide a symptom-modifying option of treatment in early OA cases (8, 9). These hemoderivatives have shown clinical improvement with no adverse effects compared with placebo in humans (48, 50) and horses (8, 9, 45), being widely used in sports medicine (45). Additionally, these treatments seem to give clients the impression of a more “natural” therapy for horses, which may be preferred over the traditional anti-inflammatory treatments. Also, both ACS and APS may offer alternatives for clinicians when other therapies are prohibited due to drug testing within equestrian competitions.

Recent studies presented in this review provided important information regarding to the lack of disease modifying effect associated with the use of these therapies. This information may help researchers direct investigations into learning how conditioned serum and protein solution composite can modulate inflammation and enhance improvement in lameness in selected cases.

The promising anecdotal results with the use of APS and the practicality of the treatment compared with ACS may lead clinicians to prefer its use (8, 48). However, both APS and ACS could benefit from additional clinical research and some refinement in product preparation and application for clinical use. The direct comparison of both products, in horses with different severities of naturally occurring OA, will further support the use of ACS and APS clinically.

## Author Contributions

LCG was responsible for the literature search and preparation of the manuscript. MJM contributed with the literature search and edition of the manuscript. All authors contributed to the article and approved the submitted version.

## Conflict of Interest

The authors declare that the research was conducted in the absence of any commercial or financial relationships that could be construed as a potential conflict of interest.

## References

[1] McIlwraith CW. Current concepts in equine degenerative joint disease. J Am Vet Med Assoc. (1982) 180:239–50. 7035425

[2] McIlwraith CW Frisbie DD Kawcak CE. The horse as a model of naturally occurring osteoarthritis. Bone Joint Res. (2012) 1:297–309. 10.1302/2046-3758.111.2000132 23610661 PMC3626203

[3] McIlwraith CW Frisbie DD Kawcak CE Fuller CJ Hurtig M Cruz A. The OARSI histopathology initiative—recommendations for histological assessments of osteoarthritis in the horse. Osteoarthritis Cartilage. (2010) 18:S93–105. 10.1016/j.joca.2010.05.031 20864027

[4] Riggs CM Whitehouse GH Boyde A. Pathology of the distal condyles of the third metacarpal and third metatarsal bones of the horse. Equine Vet J. (1999) 31:140–8. 10.1111/j.2042-3306.1999.tb03807.x 10213426

[5] USDA. National economic cost of equine lameness, colic, and equine protozoal myeloencephalitis (EPM) in the United States. In: National Animal Health Monitoring System. Washington, DC: USDA (2001).

[6] Calich ALG Domiciano DS Fuller R. Osteoarthritis: can anti-cytokine therapy play a role in treatment? Clin Rheumatol. (2010) 29:451–5. 10.1007/s10067-009-1352-3 20108016

[7] Weinberg ME Kaplan DJ Pham H Goodwin D Dold A Chiu E. Injectable biological treatments for osteoarthritis of the knee. JBJS Reviews. (2017) 5:e2. 10.2106/JBJS.RVW.16.00028 28414690

[8] Bertone AL Ishihara A Zekas LJ Wellman ML Lewis KB Schwarze RA. Evaluation of a single intra-articular injection of autologous protein solution for treatment of osteoarthritis in horses. Am J Vet Res. (2014) 75:141–51. 10.2460/ajvr.75.2.141 24471750

[9] Frisbie DD Kawcak CE Werpy NM Park RD Mcllwraith CW. Clinical, biochemical, and histologic effects of intra-articular administration of autologous conditioned serum in horses with experimentally induced osteoarthritis. Am J Vet Res. (2007) 68:290–6. 10.2460/ajvr.68.3.290 17331019

[10] Baltzer AWA Moser C Jansen SA Krauspe R. Autologous conditioned serum (Orthokine) is an effective treatment for knee osteoarthritis. Osteoarthritis Cartilage. (2009) 17:152–60. 10.1016/j.joca.2008.06.014 18674932

[11] Zarringam D Bekkers JEJ Saris DBF. Long-term effect of injection treatment for osteoarthritis in the knee by orthokin autologous conditioned serum. Cartilage. (2018) 9:140–5. 10.1177/1947603517743001 29172669 PMC5871127

[12] Kapoor M Martel-Pelletier J Lajeunesse D Pelletier JP Fahmi H. Role of proinflammatory cytokines in the pathophysiology of osteoarthritis. Nat Rev Rheumatol. (2011) 7:33–42. 10.1038/nrrheum.2010.196 21119608

[13] Kobayashi M Squires GR Mousa A Tanzer M Zukor DJ Antoniou J. Role of interleukin-1 and tumor necrosis factor α in matrix degradation of human osteoarthritic cartilage. Arthritis Rheum. (2005) 52:128–35. 10.1002/art.20776 15641080

[14] Mastbergen SC Bijlsma JWJ Lafeber FPJG. Synthesis and release of human cartilage matrix proteoglycans are differently regulated by nitric oxide and prostaglandin-E2. Ann Rheum Dis. (2008) 67:52–8. 10.1136/ard.2006.065946 17485421

[15] Coleman MC Buckwalter JA Martin JA. Potential mechanisms of PTA: oxidative stress. In: Olson S Guilak F. editors. Post-Traumatic Arthritis. Boston, MA: Springer (2015).

[16] Punzi L Galozzi P Luisetto R Favero M Ramonda R Oliviero F. Post-traumatic arthritis: overview on pathogenic mechanisms and role of inflammation. RMD Open. (2016) 2:e000279. 10.1136/rmdopen-2016-000279 27651925 PMC5013366

[17] Fernandes J Tardif G Martel-Pelletier J Lascau-Coman V Dupuis M Moldovan F. *In vivo* transfer of interleukin-1 receptor antagonist gene in osteoarthritic rabbit knee joints. Prevention of osteoarthritis progression. Am J Pathol. (1999) 154:1159–69. 10.1016/S0002-9440(10)65368-0 10233854 PMC1866546

[18] Goldring MB Berenbaum F. The regulation of chondrocyte function by proinflammatory mediators: Prostaglandins and nitric oxide. Clin Orthop Relat Res. (2004) 427:S37–46. 10.1097/01.blo.0000144484.69656.e4 15480072

[19] Smith RL Allison AC Schurman DJ. Induction of articular cartilage degradation by recombinant interleukin lα and 1β. Connect Tissue Res. (1989) 18:307–16. 10.3109/03008208909019079 2787228

[20] Frisbie DD. Autologous-conditioned serum: evidence for use in the knee. J Knee Surg. (2015) 28:63–6. 10.1055/s-0034-1543956 25599270

[21] Dinarello CA Thompson RC. Blocking IL-1: interleukin 1 receptor antagonist *in vivo* and *in vitro*. Immunol Today. (1991) 12:404–10. 10.1016/0167-5699(91)90142-G 1838480

[22] Arend WP Leung DYM. IgG Induction of IL-1 receptor antagonist production by human monocytes. Immunol Rev. (1994) 139:71–8. 10.1111/j.1600-065X.1994.tb00857.x 7927414

[23] Palmer G Guerne PA Mezin F Maret M Guicheux J Goldring MB. Production of interleukin-1 receptor antagonist by human articular chondrocytes. Arthritis Res Ther. (2002) 4:226. 10.1186/ar411 12010575 PMC111027

[24] Seitz M Loetscher P Dewald B Towbin H Ceska M Baggiolini M. Production of interleukin-1 receptor antagonist, inflammatory chemotactic proteins, and prostaglandin E by rheumatoid and osteoarthritic synoviocytes - Regulation by IFN-γ and IL-4. J Immunol. (1994) 152:2060–5. 8120407

[25] Firestein GS Boyle DL Yu C Paine MM Whisenand TD Zvaifler NJ. Synovial interleukin-1 receptor antagonist and interleukin-1 balance in rheumatoid arthritis. Arthritis Rheum. (1994) 37:644–52. 10.1002/art.1780370507 8185691

[26] Arend WP Joslin FG Massoni RJ. Effects of immune complexes on production by human monocytes of interleukin 1 or an interleukin 1 inhibitor. J Immunol. (1985) 134:3868–75. 2985700

[27] Wehling P Moser C Frisbied D McIlwraith CW Kawcak CE Krauspe R. Autologous conditioned serum in the treatment of orthopedic diseases: The Orthokine® therapy. BioDrugs. (2007) 21:323–32. 10.2165/00063030-200721050-00004 17896838

[28] Meijer H Reinecke J Becker C Tholen G Wehling P. The production of anti-inflammatory cytokines in whole blood by physico-chemical induction. Inflamm Res. (2003) 52:404–7. 10.1007/s00011-003-1197-1 14520515

[29] Frizziero A Giannotti E Oliva F Masiero S Maffulli N. Autologous conditioned serum for the treatment of osteoarthritis and other possible applications in musculoskeletal disorders. Br Med Bull. (2013) 105:169–84. 10.1093/bmb/lds016 22763153

[30] Hraha TH Doremus KM Mcilwraith CW Frisbie DD. Autologous conditioned serum: the comparative cytokine profiles of two commercial methods (IRAP and IRAP II) using equine blood. Equine Vet J. (2011) 43:516–21. 10.1111/j.2042-3306.2010.00321.x 21496084

[31] Velloso Alvarez A Boone LH Pondugula SR Caldwell F Wooldridge AA. Effects of autologous conditioned serum, autologous protein solution, and triamcinolone on inflammatory and catabolic gene expression in equine cartilage and synovial explants treated with IL-1β in Co-culture. Front Vet Sci. (2020) 7:323. 10.3389/fvets.2020.00323 32671108 PMC7332692

[32] O'Shaughnessey K Matuska A Hoeppner J Farr J Klaassen M Kaeding C. Autologous protein solution prepared from the blood of osteoarthritic patients contains an enhanced profile of anti-inflammatory cytokines and anabolic growth factors. J Orthopaedic Res. (2014) 32:1349–55. 10.1002/jor.22671 24981198 PMC4134723

[33] Muir SM Reisbig N Baria M Kaeding C Bertone AL. The concentration of plasma provides additional bioactive proteins in platelet and autologous protein solutions. Am J Sports Med. (2019) 47:1955–63. 10.1177/0363546519849671 31125271

[34] van Drumpt RAM van der Weegen W King W Toler K Macenski MM. Safety and treatment effectiveness of a single autologous protein solution injection in patients with knee osteoarthritis. BioRes Open Access. (2016) 5:261–8. 10.1089/biores.2016.0014 27668131 PMC5031090

[35] Evans CH Chevalier X Wehling P. Autologous conditioned serum. Phys Med Rehabil Clin N Am. (2016) 27:893–908. 10.1016/j.pmr.2016.06.003 27788906

[36] Rutgers M Saris DBF Dhert WJA Creemers LB. Cytokine profile of autologous conditioned serum for treatment of osteoarthritis, *in vitro* effects on cartilage metabolism and intra-articular levels after injection. Arthritis Res Ther. (2010) 12:R114. 10.1186/ar3050 20537160 PMC2911907

[37] Magalon J Bausset O Veran J Giraudo L Serratrice N Magalon G. Physico-chemical factors influencing autologous conditioned serum purification. BioRes Open Access. (2014) 3:35–8. 10.1089/biores.2013.0041 24570844 PMC3929001

[38] Takeda Y Hiraishi K Takeda H Shiobara N Shibusawa H Saniabadi AR. Cellulose acetate beads induce release of interleukin-1 receptor antagonist, but not tumour necrosis factor-α or interleukin-1β in human peripheral blood. Inflamm Res. (2003) 52:287–90. 10.1007/s00011-003-1173-9 12861393

[39] Lasarzik de Ascurra J Ehrle A Einspanier R Lischer C. Influence of incubation time and incubation tube on the cytokine and growth factor concentrations of autologous conditioned serum in horses. J Equine Vet Sci. (2019) 75:30–4. 10.1016/j.jevs.2018.12.015 31002089

[40] Gilbertie JM Davis JL Davidson GS McDonald AM Schirmer JM Schnabel LV. Oral reserpine administration in horses results in low plasma concentrations that alter platelet biology. Equine Vet J. (2018) 51:537–43. 10.1111/evj.13048 30465727

[41] Meyers KM Lindner C Katz J Grant B. Phenylbutazone inhibition of equine platelet function. Am J Vet Res. (1979) 40:265–70. 464364

[42] Yang KGA Raijmakers NJH van Arkel ERA Caron JJ Rijk PC Willems WJ. Autologous interleukin-1 receptor antagonist improves function and symptoms in osteoarthritis when compared to placebo in a prospective randomized controlled trial. Osteoarthritis Cartilage. (2008) 16:498–505. 10.1016/j.joca.2007.07.008 17825587

[43] Godek P. Use of autologous serum in treatment of lumbar radiculopathy pain. Pilot study. Ortopedia Traumatologia Rehabilitacja. (2016) 18:11–20. 10.5604/15093492.1198829 27053305

[44] Blázquez R Sánchez-Margallo FM Reinecke J Álvarez V López E Marinaro F. Conditioned serum enhances the chondrogenic and immunomodulatory behavior of mesenchymal stem cells. Front Pharmacol. (2019) 10:699. 10.3389/fphar.2019.00699 31316380 PMC6609570

[45] Ferris DJ Frisbie DD Mcilwraith CW Kawcak CE. Current joint therapy usage in equine practice: a survey of veterinarians 2009. Equine Vet J. (2011) 43:530–5. 10.1111/j.2042-3306.2010.00324.x 21668486

[46] Linardi RL Dodson ME Moss KL King WJ Ortved KF. The effect of autologous protein solution on the inflammatory cascade in stimulated equine chondrocytes. Front Vet Sci. (2019) 6:64. 10.3389/fvets.2019.00064 30895181 PMC6414419

[47] King W van der Weegen W van Drumpt R Soons H Toler K Woodell-May J. White blood cell concentration correlates with increased concentrations of IL-1ra and improvement in WOMAC pain scores in an open-label safety study of autologous protein solution. J Exp Orthop. (2016) 3:9. 10.1186/s40634-016-0043-7 26915009 PMC4747972

[48] Kon E Engebretsen L Verdonk P Nehrer S Filardo G. Autologous protein solution injections for the treatment of knee osteoarthritis: 3-year results. Am J Sports Med. (2020) 48:27–10. 10.1177/0363546520944891 32870042

[49] Wanstrath AW Hettlich BF Su L Smith A Zekas LJ Allen MJ. Evaluation of a single intra-articular injection of autologous protein solution for treatment of osteoarthritis in a canine population. Vet Surg. (2016) 45:764–74. 10.1111/vsu.12512 27391909

[50] Kon E Engebretsen L Verdonk P Nehrer S Filardo G. Clinical outcomes of knee osteoarthritis treated with an autologous protein solution injection: a 1-year pilot double-blinded randomized controlled trial. Am J Sports Med. (2018) 46:171–80. 10.1177/0363546517732734 29016185

[51] Richette P François M Vicaut E Fitting C Bardin T Corvol M. A high interleukin 1 receptor antagonist/IL-1β ratio occurs naturally in knee osteoarthritis. J Rheumatol. (2008) 35:1650–4. 18597398

[52] Lasarzik J Bondzio A Rettig M Estrada R Klaus C Ehrle A. Evaluation of two protocols using autologous conditioned serum for intra-articular therapy of equine osteoarthritis-a pilot study monitoring cytokines and cartilage-specific biomarkers. J Equine Vet Sci. (2018) 60:35–42. 10.1016/j.jevs.2016.09.014

[53] Kon E Buda R Filardo G di Martino A Timoncini A Cenacchi A. Platelet-rich plasma: intra-articular knee injections produced favorable results on degenerative cartilage lesions. Knee Surg Sports Traumatol Arthrosc. (2010) 18:472–9. 10.1007/s00167-009-0940-8 19838676

[54] van Dalen SCM Blom AB Slöetjes AW Helsen MMA Roth J Vogl T. Interleukin-1 is not involved in synovial inflammation and cartilage destruction in collagenase-induced osteoarthritis. Osteoarthritis Cartilage. (2017) 25:385–96. 10.1016/j.joca.2016.09.009 27654963

[55] Arend WP Malyak M Smith MF Whisenand TD Slack JL Sims JE. Binding of IL-1α, IL-1β, and IL-1 receptor antagonist by soluble IL-1 receptors and levels of soluble IL-1 receptors in synovial fluids. J Immunol. (1994) 153:4766–74. 7963543

[56] Chomarat P Vannier E Dechanet J Rissoan MC Banchereau J Dinarello CA. Balance of IL-1 receptor antagonist/IL-1 beta in rheumatoid synovium and its regulation by IL-4 and IL-10. J Immunol. (1995) 154:1432–9. 7822808

[57] Marques-Smith P Kallerud AS Johansen GM Boysen P Jacobsen AM Reitan KM. Is clinical effect of autologous conditioned serum in spontaneously occurring equine articular lameness related to ACS cytokine profile? BMC Vet Res. (2020) 16:181. 10.1186/s12917-020-02391-7 32513154 PMC7278142

[58] Garbin LC McIlwraith CW Frisbie DD. Evaluation of allogeneic freeze-dried platelet lysate in cartilage exposed to interleukin 1-β *in vitro*. BMC Vet Res. (2019) 15:1. 10.1186/s12917-019-2118-z 31675958 PMC6824121

